# Is CT-based body composition associated with long-term chemotherapy-induced peripheral neuropathy in colorectal cancer survivors?

**DOI:** 10.1007/s00520-022-07036-z

**Published:** 2022-04-13

**Authors:** Debbie Smit, Floortje Mols, Cynthia S. Bonhof, Martijn J. L. Bours, Gerard Vreugdenhil, Sandra Beijer

**Affiliations:** 1 CoRPS - Center of Research On Psychological Disorders and Somatic Diseases, Department of Medical and Clinical Psychology, Tilburg University, PO Box 90153, 5000 LE Tilburg, The Netherlands; 2 Department of Research & Development, Netherlands Comprehensive Cancer Organisation (IKNL), Utrecht, The Netherlands; 3 Department of Epidemiology, GROW – School for Oncology & Developmental Biology, Maastricht University, Maastricht, The Netherlands; 4 Department of Internal Medicine, Máxima Medical Centre, Eindhoven and Veldhoven, The Netherlands

**Keywords:** Adipose tissue, Body composition, Chemotherapy-induced peripheral neuropathy, Colorectal cancer, Computed tomography, Skeletal muscle density, Skeletal muscle mass

## Abstract

**Background:**

Chemotherapy-induced peripheral neuropathy (CIPN) is a common side effect among colorectal cancer (CRC) survivors, and the severity is mainly dependent on the chemotherapy dose. Nowadays, chemotherapy dose is based on body surface area, while determination based on more accurate measures of body composition may be better. This study aimed to investigate the association between body composition and long-term CIPN among CRC survivors 2–11 years after diagnosis.

**Methods:**

Data from CRC survivors from the population-based PROFILES registry were used. Survivors were included when they received chemotherapy, filled in the EORTC QLQ-CIPN20, and had a computed tomography (CT) scan at diagnosis (*n* = 202). Total, sensory, motor, and autonomic CIPN were based upon the EORTC QLQ-CIPN20. The abdominal CT scans were used to determine skeletal muscle index (SMI), skeletal muscle density (SMD), visceral adipose tissue (VAT), subcutaneous adipose tissue (SAT), and total adipose tissue (TAT). Logistic regression was used to analyze the association between CIPN outcomes and body composition variables.

**Results:**

CIPN was experienced by 64% of the CRC survivors several years after chemotherapy. More SAT was associated with a higher odds of reporting total CIPN (OR = 1.01 95% CI 1.00–1.01, *p* = 0.01), motor CIPN (OR = 1.01 95% CI 1.00–1.01, *p* = 0.01), and sensory CIPN (OR = 1.01 95% CI 1.00–1.01, *p* = 0.04). No associations of other body composition parameters with CIPN were observed.

**Conclusion:**

Only SAT was associated with total, motor, and sensory CIPN. Based on these results, we cannot conclude that determining the chemotherapy dose based on body composition is preferred over determining the chemotherapy dose based on body surface to prevent CIPN. More research is needed to assess associations of body composition with CIPN, a common side effect of chemotherapy.

## Introduction

There is an increasing number of colorectal cancer (CRC) survivors worldwide [1]. In 2020, CRC was the third most common cancer with 10% of all new cancer cases and more than 1.9 million incident cases [2]. As a result, the number of people dealing with the side effects of CRC and its treatment is growing. Besides surgery, a common (neo)adjuvant treatment for CRC is chemotherapy [3]. Chemotherapy-induced peripheral neuropathy (CIPN) is a common and disabling side effect of various types of chemotherapy, like oxaliplatin [3]. CIPN can be an acute and a chronic side effect that can last for years and negatively impacts quality of life [3–6]. CIPN can be divided into three components, in which symptoms of sensory neuropathy consist of, for example, tingling and pain in the hands or feet; motor neuropathy consists of cramps and problems with writing; and autonomic neuropathy involves problems with vision and dizziness [7].

The severity of CIPN is mainly dependent on the cumulative dose of the administered chemotherapy. Because there are very few options for the treatment of CIPN, it is important to determine the optimal chemotherapy dose to try to prevent CIPN [3, 8, 9]. Nowadays, the chemotherapy dose is often based on body surface area (estimated from body height and weight) where body composition, the distribution of muscle and fat mass, and pharmacokinetics are not taken into account [10, 11]. It is known that people with the same body surface can differ in body composition, resulting in variations in the drug efficacy and concentrations [10]. For example, oxaliplatin is a lipophilic drug which can build up in the fat tissue. Patients with less skeletal muscle mass and more fat mass may have higher concentrations of the drug. This can possibly lead to higher toxicity including more and severe CIPN complaints, both acute and chronic [3, 8, 9, 12–15]. Some literature suggests that it might be better to determine the chemotherapy dose based on body composition rather than body surface [10–16].

In patients with cancer, skeletal muscle mass and skeletal muscle density (SMD) often decline due to the tumor and/or its treatment while adipose tissue can both increase and decrease [10, 11, 13, 15, 17]. SMD reflects the lipid content of the muscle cells; lower SMD is associated with higher muscle cell lipid content [18]. Studies show that at diagnosis, about 30–40% of CRC patients already have low SMD, 43% had low SMI, and 64% have a high percentage of visceral adipose tissue (VAT) [19–22]. A study in patients with different tumor types showed that a low muscle mass was associated with more chemotoxicity. In the same study, VAT and subcutaneous adipose tissue (SAT) were not associated with more chemotoxicity [23].

To the best of our knowledge, no studies have investigated the specific association between body composition and chronic CIPN. Most studies explored the association between chemotherapy dose (calculated upon body surface) and chemotoxicity. Concerning CIPN, it may be a better option to look at body composition when determining the dose of chemotherapy [11]. Therefore, in the current study, we investigated the association between body composition (SMI, SMD, VAT, SAT, and total adipose tissue (TAT)) and long-term CIPN among CRC survivors 2 to 11 years after diagnosis. It is hypothesized that CRC patients with a lower muscle mass, lower SMD, and more adipose tissue at diagnosis will more often report chronic CIPN.

## Methods

### Setting and participants

For the current study, data were used from a longitudinal, population-based cohort study among CRC survivors (stages I–IV) who were diagnosed between 2000 and 2009 in the southern part of the Netherlands. The patients were selected from the Netherlands Cancer Registry (NCR), which is responsible for the clinical data collection of all newly diagnosed cancer patients in the Netherlands [24]. Data collection was conducted in PROFILES, which was previously described in more detail [4, 25]. In brief, data collection started in December 2010 when CRC survivors were recruited by a letter from their (ex-attending) specialist to inform them about the study and were asked to participate. Within 2 months, non-respondents received a reminder. After giving informed consent, participants received a digital or paper and pencil questionnaire yearly until 2013. Exclusion criteria for the cohort study were unverifiable addresses, having cognitive impairment, those who died before the start of the study or were terminally ill, had stage 0 disease/carcinoma in situ, or those already included in another PROFILES study.

The study was conducted according to the Declaration of Helsinki guidelines, and ethical approval for the study was obtained from the certified medical ethics committee of the Maxima Medical Centre in the Netherlands.

The current study used data of the second data collection wave which was 1 year after inclusion (December 2011), because in this second wave, CIPN was assessed. Only CRC survivors who received chemotherapy, of whom a computed tomography (CT) scan of good quality was available, and who filled in a CIPN questionnaire were included in the analyses.

### Measurements

#### Sociodemographic and clinical characteristics

Sociodemographic characteristics were derived from the questionnaire and included sex, age, educational level, marital status, and employment status. The presence of comorbid conditions was assessed with the adjusted Self-Administered Comorbidity Questionnaire [26]. Clinical characteristics of the CRC diagnosis, including tumor type, time since diagnosis, treatment (surgery, radiotherapy, chemotherapy), and cancer stage were retrieved from the NCR [24].

#### Peripheral neuropathy

The European Organization for Research and Treatment of Cancer Quality of Life Questionnaire Chemotherapy-Induced Peripheral Neuropathy 20 (EORTC QLQ-CIPN20) was used to assess CIPN [7]. This questionnaire consists of three subscales: sensory, motor, and autonomic CIPN. In this questionnaire, patients are asked how often they experienced a neuropathy symptom in the past week. The items are answered on a 4-point Likert scale ranging from (1) not at all to (4) very much. These scores were linearly transformed to a 0–100 scale in which higher scores mean more neuropathy complaints.

A total score was also calculated according to standard EORTC scoring procedures for symptom scales. Questions 19 and 20 (on driving cars and getting an erection) were excluded in calculating the sum score, because these items are not relevant for everyone. A raw mean score was calculated of the other 18 items and transformed to a 0–100 scale in which higher scores indicate more complaints. The total and subscale scores (sensory, autonomic, motor CIPN) were, with help of sex and age-specific cutoff values, dichotomized into yes/no CIPN. The used sex and age-specific cutoff points were based on a previous study [27] (Table 1). When half of the questions were missing in calculating the subscale or total score, the corresponding score was considered missing.

**Table 1:** Cutoff points of chemotherapy-induced peripheral neuropathy (CIPN) for the results of the EORTC QLQ-CIPN20 questionnaire [27]

	Men				Women			
Age (years)	40–49	50–59	60–69	70–92	40–49	50–59	60–69	70–92
Total CIPN	1.8	2.1	2.8	6.4	3.3	4.7	4.6	8.4
Sensory CIPN	1.8	2.2	2.9	6.8	2.6	4.1	3.5	7.3
Motor CIPN	1.7	2.0	2.3	6.3	3.6	5.5	6.2	10.2
Autonomic CIPN	2.5	2.8	4.1	5.4	4.9	4.7	4.1	6.9

#### Body composition

Abdominal CT scans routinely performed at diagnosis were used to assess body composition. These CT scans were analyzed by trained researchers following standardized procedures. Skeletal muscle, VAT, and SAT cross-sectional areas were quantified on CT images at the level of the 3rd lumbar vertebrae with the use of Slice-O-Matic software version 5.0 (Tomovision, Montreal, Canada). To identify the different tissues, standard density thresholds, measured in Hounsfield units (HU) were used.

The standard density threshold for skeletal muscle mass was − 29 to 150 HU, for VAT − 150 to − 50 HU, and for SAT and intermuscular adipose tissue (IMAT) − 190 and − 30 HU. Total adipose tissue (TAT) (cm^2^) was calculated as the sum of VAT, SAT, and IMAT (cm^2^). SMD was assessed as the mean radiographic density (HU) of the total skeletal muscle cross-sectional area. To adjust for height, the skeletal muscle mass index (SMI) (cm^2^/m^2^) was calculated by dividing the skeletal muscle cross-sectional area (cm^2^) by squared height (m^2^). Body mass index (BMI) (kg/m^2^) was calculated by dividing body weight (kg) by squared height (m^2^). Body height and weight were self-reported.

### Statistical analyses

Differences in sociodemographic and clinical characteristics between patients with and without total CIPN, sensory CIPN, motor CIPN, and autonomic CIPN and between included and excluded patients (patients without a CT scan of good quality) were examined with independent *t*-tests for continuous variables and chi-square tests for categorical variables.

Logistic regression was used to analyze the association between the four dichotomized neuropathy outcomes (i.e., total CIPN, sensory CIPN, motor CIPN, and autonomic CIPN) and the body composition variables (i.e., SMD, SMI, SAT, VAT, and TAT). The adjusted model included a priori determined confounding factors based on literature, including time since diagnosis, tumor type (colon or rectal cancer), sex, and age. Other potential confounders, i.e., SMD and SMI (in the VAT, SAT, and TAT analyses), SAT (in the SMD, SMI, and VAT analyses), VAT (in the SMD, SMI, and SAT analyses), and TAT (in the SMI and SMD analyses), were included in the final models if they changed the OR for CIPN with at least 10% when the variable was added to the model [20, 21]. This was not the case for any of these body composition variables. Therefore, the adjusted models only included time since diagnoses, tumor type, sex, and age. Interaction between body composition and sex was also explored by including interaction terms in the logistic models. When the interaction term between the different body composition components and sex was significant (*p* < 0.1) in the final model, the analysis was stratified for sex.

Analyses were performed using SAS software (version 9.4, SAS Institute Inc., Cary, NC, USA). *P*-values < 0.05 were considered statistically significant.

## Results

### Sociodemographic and clinical characteristics

Data of 1684 respondents were available from the second wave of the cohort study. CT scans of 582 CRC survivors were available, of whom 205 received chemotherapy. Neuropathy data was missing for three CRC survivors, which yielded a total of 202 CRC survivors in the final analysis.

Of the 202 survivors, 64% (*n* = 129, 1 missing) reported total CIPN above the sex and age-specific cutoff values (Table 2). CRC survivors with CIPN were significantly younger, had a shorter time since diagnosis, had more SAT, and received radiotherapy more often compared to survivors without CIPN.

**Table 2:** Sociodemographic and clinical characteristics of colorectal cancer patients, stratified by total chemotherapy-induced peripheral neuropathy (CIPN)

	Total group (*n* = 202)	No total CIPN^a^ (*n* = 72)	Total CIPN^a^ (*n* = 129)	*P*-value
Age questionnaire (years, mean (SD))	66 (9.9)	68 (8.5)	65 (10.1)	0.01
Sex (female, *n* (%))	93 (46)	38 (53)	58 (45	0.76
Married/cohabited (yes, *n* (%))	167 (83)	57 (80)	110 (85)	0.36
Educational level Low Middle High	25 (12) 129 (64) 47 (23)	8 (11) 45 (63) 19 (26)	17 (13) 83 (65) 28 (22)	0.74
Employed (yes, *n* (%))	46 (23)	15 (21)	31 (25)	0.58
BMI (kg/m^2^, mean (SD))	26.5 (3.5)	26.1 (3.2)	26.7 (3.7)	0.19
Comorbidities (*n* (%)) 0 1 ≥ 2	52 (28) 70 (37) 66 (35)	20 (29) 28 (41) 20 (29)	32 (27) 41 (34) 46 (39)	0.43
Tumor location (*n* (%)) Colon Rectal	126 (63) 75 (37)	39 (54) 33 (46)	86 (67) 42 (33)	0.08
Time since diagnosis (years, mean (SD))	4.2 (1.84)	4.6 (2.01)	4.0 (1.72)	0.03
Stage (*n* (%)) I II III IV Unknown	12 (6) 25 (12) 135 (67) 21 (10) 9 (4)	4 (6) 11 (15) 48 (67) 4 (6) 5 (7)	8 (6) 13 (10) 87 (67) 17 (13) 4 (3)	0.27
Surgery (yes, *n* (%))	199 (99)	70 (97)	128 (99)	0.26
Radiotherapy (yes, *n* (%))	68 (36)	32 (44)	35 (27)	0.01
Total CIPN^a^ (missing = 1) Mean (SD) Yes (*n* (%))	11 (12.8) 129 (64)	2 (1.9)	17 (13.3)	< 0.001
Motor CIPN (missing = 1) Mean (SD) Yes (*n* (%))	10 (14.7) 99 (49)	2 (2.9) 4 (6)	15 (16.5) 95 (74)	< 0.001 < 0.001
Sensory CIPN Mean (SD) Yes (*n* (%))	12 (14.7) 132 (65)	2 (2.7) 12 (17)	18 (15.2) 120 (93)	< 0.001 < 0.001
Autonomic CIPN Mean (SD) Yes (*n* (%))	7 (13.5) 58 (29)	2 (5.3) 8(11)	10 (15.6) 50 (39)	< 0.001 < 0.001
Muscle mass (cm^2^, mean (SD))	140 (32.7)	138 (31.9)	141 (33.2)	0.55
SMD (hu, mean (SD))	38 (8.0)	38 (6.8)	38 (8.6)	0.98
SMI (cm^2^/m^2^, mean (SD))	47 (8.5)	47 (8.4)	47 (8.6)	0.85
SAT (cm^2^, mean (SD))	156 (77.9)	137 (85.3)	167 (71.9)	0.01
VAT (cm^2^, mean (SD))	132 (91.2)	128 (93.4)	134 (90.5)	0.66
TAT (cm^2^, mean (SD))	301 (135.8)	277 (142.5)	314 (131.1)	0.06

^a^Based on the sum score of the EORTC QLQ-CIPN20[27]

Abbreviations: *CIPN* chemotherapy-induced peripheral neuropathy, *SMD* skeletal muscle density, *SMI* skeletal muscle mass index

Looking at the total included group, 65% of the CRC survivors reported sensory CIPN (*n* = 132), 49% motor CIPN (*n* = 99), and 29% autonomic CIPN (*n* = 58). CRC survivors with sensory CIPN had a shorter time since diagnosis (4.0 vs. 4.6 years, *p* = 0.03), underwent surgery more often (66% vs. 34%, *p* = 0.02), and had more SAT (164.1 vs 141.2 cm^2^, *p* = 0.046) compared with CRC survivors without sensory CIPN. CRC survivors with motor CIPN had a shorter time since diagnosis (3.9 vs. 4.6 years, *p* = 0.04) and had more SAT (172.0 vs. 141.0 cm^2^, *p* < 0.01) compared with CRC survivors without motor CIPN. No differences were found in sociodemographic or clinical characteristics between survivors with and without autonomic CIPN. In addition, sociodemographic and clinical characteristics were also not different between the included and excluded survivors (survivors without a CT scan of good quality) (data not shown).

### Skeletal muscle mass and skeletal muscle density

No significant associations were found between SMD or SMI and total CIPN. Also, no significant associations were found for SMD or SMI with motor, sensory, or autonomic CIPN (Table 3). The interaction terms for SMI or SMD and sex were not significant for all the CIPN outcomes.

**Table 3:** Association between SMD, SMI, and chemotherapy-induced peripheral neuropathy (CIPN)

	Total CIPN^a,b^		Motor CIPN^b^			Sensor CIPN^b^		Autonomic CIPN^b^	
	OR (95% CI)	*p*	OR (95% CI)	*p*		OR (95% CI)	*p*	OR (95% CI)	*p*
SMD (hu)									
Unadjusted	1.00 (0.96–1.04)	0.99	1.00 (0.97–1.04)	0.85		0.98 (0.95–1.01)	0.26	1.01 (0.98–1.05)	0.48
Adjusted	0.99 (0.94–1.03)	0.53	1.00 (0.95–1.04)	0.94		0.97 (0.93–1.01)	0.16	1.01 (0.96–1.06)	0.72
SMI (cm^2^/m^2^)									
Unadjusted	1.00 (0.97–1.04)	0.85	1.00 (0.97–1.04)	0.92		1.00 (0.96–1.03)	0.79	0.99 (0.95–1.03)	0.55
Adjusted	0.98 (0.93–1.04)	0.55	0.99 (0.94–1.04)	0.63		0.99 (0.96–1.03)	0.69	0.98 (0.93–1.03)	0.41

Abbreviations: *CIPN* chemotherapy-induced peripheral neuropathy, *SMD* skeletal muscle density, *SMI* skeletal muscle mass index

^a^Based on the sum score of the EORTC QLQ-CIPN20[27]

^b^Adjusted for time since diagnoses, age, tumor type (colon/rectum), and sex

### Fat mass

The interaction terms between sex and SAT, VAT, or TAT were not significant in any of the models. Therefore, the models were not stratified for sex. More SAT (cm^2^) was associated with a higher odds of reporting total CIPN (OR = 1.01 95% CI 1.00–1.01 *p* = 0.01), sensory CIPN (OR = 1.01 95% CI 1.00–1.01 *p* = 0.04), and motor CIPN (OR = 1.01 95% CI 1.00–1.01, *p* = 0.01. No significant associations were found between SAT and autonomic CIPN or between VAT and TAT and any of the CIPN outcomes (Table 4).

**Table 4:** Association between fat tissue and chemotherapy-induced peripheral neuropathy (CIPN)

	Total CIPN^a,b^		Motor CIPN^b^		Sensory CIPN^b^		Autonomic CIPN^b^	
	OR (95% CI)	*p*		*p*	OR (95% CI)	*p*	OR (95% CI)	*p*
SAT (cm^2^)								
Unadjusted	1.01 (1.00–1.02)*	0.01*	1.01 (1.00–1.01)	0.01*	1.00 (1.00–1.01)	0.05	1.00 (1.00–1.00)	0.81
Adjusted	1.01 (1.00–1.01)*	0.01*	1.01 (1.00–1.01)	0.01*	1.01 (1.00–1.01)*	0.04*	1.00 (1.00–1.00)	0.82
VAT (cm^2^)								
Unadjusted	1.00 (1.00–1.00)	0.65	1.00 (1.00–1.00)	0.88	1.00 (1.00–1.00)	0.55	1.00 (1.00–1.00)	0.74
Adjusted	1.00 (1.00–1.01)	0.70	1.00 (1.00–1.00)	0.97	1.00 (1.00–1.01)	0.47	1.00 (1.00–1.01)	0.48
TAT (cm^2^)								
Unadjusted	1.00 (1.00–1.00)	0.06	1.00 (1.00–1.00)	0.11	1.00 (1.00–1.00)	0.11	1.00 (1.00–1.00)	0.76
Adjusted	1.00 (1.00–1.00)	0.08	1.00 (1.00–1.00)	0.12	1.00 (1.00–1.01)	0.15	1.00 (1.00–1.00)	0.61

Abbreviations: *CIPN* chemotherapy-induced peripheral neuropathy, *SAT* subcutaneous adipose tissue, *VAT* visceral adipose tissue, *TAT* total adipose tissue

^a^Based on the sum score of the EORTC QLQ-CIPN20[27]

^b^Adjusted for time since diagnoses, age, tumor type (colon/rectum), and sex

^*^Significant

## Discussion

In this study, the association between CT-based body composition and CIPN in CRC survivors 2–11 years after diagnosis was assessed. Results showed that 64% of the CRC survivors experienced total CIPN several years after chemotherapy. Furthermore, it was showed that more SAT was associated with a higher odds of reporting total CIPN, sensory CIPN, and motor CIPN. However, an association of VAT, TAT, SMI, and SMD with CIPN was not found.

The findings of the current study regarding the association between fat mass and CIPN are only partly in line with results of previous studies. In our study, an association was found between SAT and total, sensory, and motor CIPN. This is in contrast with a study among patients with different types of cancers, including CRC, in which no association between SAT and chemotoxicity was found [23]. However, in that study, no association was found between VAT and chemotoxicity, and we also observed no association between VAT or TAT and total, sensory, and motor CIPN. A review also showed no conclusive results between fat mass and chemotoxicities [15]. However, in that review, only one study was included which studied the effect of oxaliplatin in CRC patients [15]. As said before, oxaliplatin is a lipophilic drug often used in the treatment of CRC which can build up in the fat tissue. Patients with more fat mass, including SAT, may have higher concentrations of the drug [3, 8, 9, 12–15], which might explain the association we found between more SAT and more often reported CIPN.

In our study, no association was found between SMI or SMD and total, motor, sensory, or autonomic CIPN. These results were not in line with a study which showed that a low muscle mass was associated with more chemotoxicity [23]. Furthermore, a study in esophagogastric cancer patients showed that patients with sarcopenic obesity (sarcopenia with overweight or obesity (BMI > 25 kg/m^2^)) before chemotherapy had a higher risk of sensory CIPN [13]. A reason why we did not find an association between VAT, TAT, SMI or SMD, and CIPN might be the lack of CT scans just before chemotherapy. In patients with colon cancer, chemotherapy is given after surgery. In the period between diagnosis and the start of the chemotherapy, skeletal muscle mass may further decline. It might be possible that at time of diagnosis, skeletal muscle mass was significantly higher compared to the skeletal muscle mass just before chemotherapy, which could have led to the non-significant findings regarding its association with sensory neuropathy.

This study has a few limitations that need to be mentioned. First, neuropathy was assessed once, on average 6 years after diagnosis. Therefore, the change in neuropathy before, during, and after chemotherapy cannot be assessed. It might be possible that the patients already had neuropathy symptoms before chemotherapy treatment, so it is not known which percentage of acute neuropathy complaints is due to the chemotherapy. Furthermore, we had no information on the cumulative dose of the received chemotherapy. Therefore, we cannot say something about the relation between body composition, received chemotherapy dose, and CIPN complaints. Related to this, we also had no information on adjustments of chemotherapy dosage because of neuropathy complaints. If the chemotherapy dose was determined based on body surface and overestimated, adjustments of the dose during the chemotherapy cycles might have prevented chronic neuropathy. Another limitation is that only information about body composition at diagnosis was available. Because body composition was not measured over time, we do not know whether body composition changed after diagnosis and during surgery and chemotherapy, which might have influenced the association. Another limitation can be the low cutoff points for CIPN (Table 1), resulting in a combination of survivors with few CIPN complaints and survivors with more complaints in the same group. However, when higher cutoff points were used, groups would have become too small, and mild symptoms of neuropathy were not taken into account. Taking mild symptoms into account is important since this can have a negative impact on quality of life years after treatment [3–6]. Furthermore, the cutoff points were based on a study in a similar population, using the same cutoff points makes this study more comparable [27].

Despite these limitations, this study also has some meaningful strengths. An important strength of this study is the use of CT scans to assess the body composition of the patients. CT scans are a non-invasive way to determine the cross-sectional areas of skeletal muscle and adipose tissue [28] and are part of the standard diagnostic procedures in colorectal cancer. To guarantee reliability, CT scans were analyzed by trained researchers following standardized procedures. Another important strength of this study is that we had usable CT scans available of quite a large group of CRC survivors which provided reliable information about body composition. While we did not have CT scans from all patients, the group with and without CT scans had comparable sociodemographic and clinical characteristics, and therefore, it is not likely that this influenced our results. Furthermore, this was the first large study which investigated the association between body composition and long-term CIPN.

In conclusion, results showed that 64% of the CRC survivors experienced persistent CIPN complaints several years after chemotherapy. It was showed that more SAT was associated with a higher odds of reporting total CIPN, sensory CIPN, and motor CIPN 2–11 years after diagnosis. No associations were found between TAT, VAT, SMI or SMD, and CIPN. Because CIPN is a severe and disabling side effect of chemotherapy, insight in how to prevent CIPN is important. However, based on our results, we cannot conclude that determining the chemotherapy dose based on body composition is preferred over determining the chemotherapy dose based on body surface to prevent CIPN. Large longitudinal observational studies that assess chemotherapy dosing and adjustments CIPN, and body composition at multiple time points from diagnosis, during treatment until years later, are needed to shed light into this understudied area.

## Data Availability

The datasets generated during and/or analyzed during the current study, excluding the CT scans, are available from the corresponding author on reasonable request.

## Abbreviations

CIPN: Chemotherapy-induced peripheral neuropathy
CRC: Colorectal cancer
CT: Computed tomography
PN: Peripheral neuropathy
NCR: Netherlands Cancer Registry
SMI: Skeletal muscle index
SAT: Subcutaneous adipose tissue
SMD: Skeletal muscle density
TAT: Total adipose tissue
VAT: Visceral adipose tissue
